# Axial and Nonaxial Migration of Red Blood Cells in a Microtube

**DOI:** 10.3390/mi12101162

**Published:** 2021-09-28

**Authors:** Naoki Takeishi, Hiroshi Yamashita, Toshihiro Omori, Naoto Yokoyama, Masako Sugihara-Seki

**Affiliations:** 1 Graduate School of Engineering Science, Osaka University, 1-3 Machikaneyama, Toyonaka 560-8531, Japan; yamashita@biomech.me.es.osaka-u.ac.jp (H.Y.); sekim@kansai-u.ac.jp (M.S.-S.); 2 Department of Pure and Applied Physics, Kansai University, 3-3-35 Yamate-cho, Suita 564-8680, Japan; 3 Department of Finemechanics, Tohoku University, 6-6-01 Aoba, Sendai 980-8579, Japan; toshihiro.omori.b5@tohoku.ac.jp; 4 Department of Mechanical Engineering, Tokyo Denki University, 5 Senju-Asahi, Adachi, Tokyo 120-8551, Japan; n.yokoyama@mail.dendai.ac.jp

**Keywords:** red blood cells, axial migration, lattice-Boltzmann method, finite element method, immersed boundary method, computational biomechanics

## Abstract

Human red blood cells (RBCs) are subjected to high viscous shear stress, especially during microcirculation, resulting in stable deformed shapes such as parachute or slipper shape. Those unique deformed RBC shapes, accompanied with axial or nonaxial migration, cannot be fully described according to traditional knowledge about lateral movement of deformable spherical particles. Although several experimental and numerical studies have investigated RBC behavior in microchannels with similar diameters as RBCs, the detailed mechanical characteristics of RBC lateral movement—in particular, regarding the relationship between stable deformed shapes, equilibrium radial RBC position, and membrane load—has not yet been fully described. Thus, we numerically investigated the behavior of single RBCs with radii of 4 μm in a circular microchannel with diameters of 15 μm. Flow was assumed to be almost inertialess. The problem was characterized by the capillary number, which is the ratio between fluid viscous force and membrane elastic force. The power (or energy dissipation) associated with membrane deformations was introduced to quantify the state of membrane loads. Simulations were performed with different capillary numbers, viscosity ratios of the internal to external fluids of RBCs, and initial RBC centroid positions. Our numerical results demonstrated that axial or nonaxial migration of RBC depended on the stable deformed RBC shapes, and the equilibrium radial position of the RBC centroid correlated well with energy expenditure associated with membrane deformations.

## 1. Introduction

The flow behavior of human red blood cells (RBCs) in capillaries has been intensively studied from various points of view, including shape, deformability, and physiological functions such as oxygen transport. Due to the large number of blood cells, the temporal shapes of individual RBCs during flow are of fundamental importance both in micro- and macro-scale hemorheology [1,2,3]. Especially in the microcirculation, where flow can be assumed to be almost inertialess, RBCs are subjected to high viscous shear stress, resulting a bistable shape ( parachute or slipper shape) in capillaries [4,5,6]. Recent numerical analysis further showed that the asymmetrical slipper shape of RBCs was observed not only in circular microchannels, but also in narrow rectangular microchannels, whose width was comparable to the thickness of an RBC [7]. The unique flow behavior of RBCs, which is often accompanied by axial or nonaxial migration, cannot be described using the traditional framework regarding lateral movement of deformable spherical particles, as originally reported in [8]. In this framework, a deformable spherical particle tends to move toward the channel axis and remains there. Assuming that the stable shape of RBCs under flow results from the force balance between internal/external hydrodynamic shear force and inner elastic force on the membrane, model analysis will provide insight into the mechanical background of both stable shape and equilibrium radial position.

So far, the impact on the stable RBC shape of various mechanical conditions, including flow speed, membrane elasticity, channel confinement, and cell volume–surface ratio, has been investigated using a 2D elastic spring model [9], a 2D vesicle model [1,2,10,11], 3D models such as vesicle model [12], a mesoscopic molecular dynamics model [13], and a capsule model [5,6]. These investigations showed that RBCs tend to form a stable parachute shape in a circular microchannel with a diameter comparable to that of an RBC (≤10 μm) [6,12,13]. This shape was also observed in a narrow rectangular microchannel with a width comparable to the thickness of an RBC ≤ 3.5 μm [7]. In a slightly larger microchannel (≥12 μm), shape bistability became significant [5,13]. Guckenberger et al. (2018) further showed the effect of the initial position of the RBC centroid on the stable deformed configuration in Stokes flow for different capillary numbers Ca, which defines the ratio between a fluid viscous force and a membrane elastic force [5]. Despite these efforts, the relationships between those stable deformed shapes, the equilibrium radial positions of RBCs, and the membrane load have not yet been fully described.

Along with the aforementioned numerical studies, recent microfluidic techniques have allowed us to conduct high-throughput measurements of single-cell behavior under confined channel flow [14,15,16,17]. Since the stable shape of an RBC under flow is highly reliant on cell mechanical properties, quantifying cell shapes in microfluidic systems will be useful for understanding cell states—including the ability of RBCs to function as oxygen carriers—and might be extended to the diagnosis of blood diseases [18,19]. In particular, patients with sickle cell anemia have a high hemoglobin concentration that results in abnormal rheology [20,21,22]. Hence, alternations in membrane elasticity and the relationship between viscosity ratio and the steady shape of RBCs are clinically important. If the stable shape of RBCs is changed depending on viscosity ratios of the internal to external fluids of RBCs, the shape would be a hallmark to identify the cell state, or be useful in cellular-level diagnoses for blood diseases.

Therefore, the objective of this study is to reveal the relationship between stable deformed RBC shapes, their equilibrium radial positions, and the membrane loads of flowing RBCs in a microtube. We numerically investigated the behavior of a single RBC with a major diameter of 8 μm in a straight circular microchannel with 15 μm-diameter. The RBC was modeled as a biconcave capsule, whose membranes followed the Skalak constitutive (SK) law [23]. Internal and external fluids were modeled as an incompressible, Newtonian viscous fluid. The problem was characterized by Reynolds number and the capillary number Ca. The flow was assumed to be almost inertialess. The power (or energy dissipation) associated with membrane deformations was considered to quantify the state of membrane loads. Simulations were performed for different capillary numbers and viscosity ratios, as well as different initial positions of the RBC centroid.

## 2. Methods

### 2.1. Flow and RBC Model

**Figure 1:**
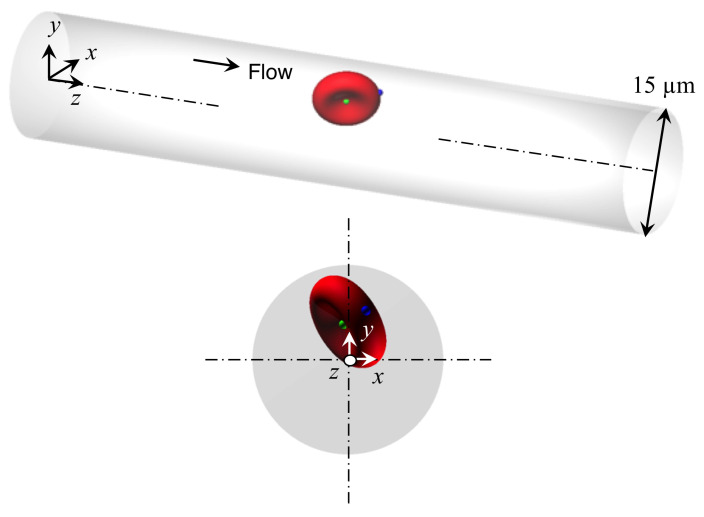
Simulation setup: A single RBC with radius of 4 μm is placed with random orientation in a circular channel with diameter of 15 μm and length of 80 μm. Periodic boundary conditions are imposed on the flow (*z*-direction) and no-slip conditions are employed for the wall (*x*- and *y*-direction). Green dots represent material points at the initial concave node point, and blue dots at the initial edge node point.

We consider a cellular flow consisting of an external fluid (plasma), internal fluid (cytoplasm), and RBC with radius *a* in a circular channel of diameter *D* (2*R*), with a resolution of 16 fluid lattices per radius of RBC. This resolution was also applied in studies of channel flows [24,25] and the rheology of RBC suspensions [3]. The channel length is set to be 20*a*. The same computational length was also applied in the numerical analysis in [13]. In this study, we focused on the transition of RBC shape, especially from parachute shape to slipper shape [5,13], which usually occurs for D≥ 12 μm assuming *a* = 4 μm [5,13,24]. Thus, we set the channel diameter to be *D* = 15 μm. The RBC is modeled as a biconcave capsule, or a Newtonian fluid enclosed by a thin elastic membrane, with a major diameter *d* = 8 μm (2*a*) and maximum thickness 2 μm (*a*/2) [26]. The RBC is placed in a computational domain and is shown in Figure 1, where material points at the initial concave node point are represented by green dots, and those at the initial edge node point are indicated by blue dots.

The membrane is modeled as an isotropic and hyperelastic material following the SK law [23]. The strain energy *w* of the SK law is given by

(1) $$w=\frac{G_{s}}{4}\left(I_{1}^{2}+2I_{1}-2I_{2}+CI_{2}^{2}\right),$$

where $G_{s}$ is the surface shear elastic modulus; *C* is a coefficient representing the area incompressibility; $I_{1}$ ($\lambda_{1}^{2}+\lambda_{2}^{2}-2$) and $I_{2}$ ($\lambda_{1}^{2}\lambda_{2}^{2}-1=J_{s}^{2}-1$) are the first and second invariants of the Green–Lagrange strain tensor; $\lambda_{i}$ (*i* = 1 and 2) are the two principal in-plane stretch ratios; and $J_{s}=\lambda_{1}\lambda_{2}$ is the Jacobian, which expresses the ratio of the deformed to reference surface areas. In this study, we set $C=10^{2}$ [27]. Bending resistance was also considered [28], with a bending modulus $k_{b}=5.0\times 10^{-19}$ J [29]. By mimicking a previous stretch experiment involving RBCs [30], the surface shear elastic modulus and the area incompressibility coefficient of RBCs at Re = 0.2 were determined to be $G_{s}$ = 4.0 μN/m and *C* = 10${}^{2}$, respectively [3,24]. These membrane parameters successfully reproduced the deformation of RBCs in shear flow [3,24] and also the thickness of the cell-depleted peripheral layer in circular channels [24]. We define the initial shape of RBCs as biconcave.

Neglecting inertial effects on membrane deformation, the static local equilibrium equation of the membrane is given by

(2) $$\nabla_{s}\cdot T+q=0,$$

where $\nabla_{s}\left(\left(I-nn\right)\cdot \nabla \right)$ is the surface gradient operator, n is the unit normal outward vector in the deformed state, and T is the in-plane elastic tension that is obtained from the SK law (1).

It is known that the usual distribution of hemoglobin concentration in individual RBCs ranges from 27 to 37 g/dL corresponding to the internal fluid viscosity being $\mu_{1}$ = 5–15 cP (5–15 mPa·s) [31], while the normal plasma viscosity is $\mu_{0}$ = 1.1 − 1.3 cP (1.1 − 1.3 mPa·s) for plasma at 37 ℃ [32]. Hence, the physiologically relevant viscosity ratio can be taken as $\lambda (\mu_{1}/\mu_{0})$ = 4.2 − 12.5 if the plasma viscosity is set to be $\mu_{0}$ = 1.2 cP. Hence, in our study, the physiological relevant viscosity ratio is set to be λ = 5−10. The fluids are modeled as an incompressible Navier–Stokes equation, with a governing equation of fluid velocity v:

(3) $$\begin{array}{cc} \rho \left(\frac{\partial v}{\partial t}+v\cdot \nabla v\right) & =-\nabla p-\nabla \cdot \tau +\rho f, \end{array}$$

(4) $$\begin{array}{cc} \nabla \cdot v & =0, \end{array}$$

where *p* is the pressure, ρ is the fluid density, f is the body force, and τ is the stress tensor of liquids and can be expressed by

(5) $$\begin{array}{cc} \tau \; & =-\mu \left(\nabla v+\nabla v^{T}\right),\\ \; & =-\left\{\left(1-\alpha \right)\mu_{0}+\alpha \mu_{1}\right\}\left(\nabla v+\nabla v^{T}\right), \end{array}$$

where α is volume fraction of the inner fluid, which is in the range of 0 ≤α≤ 1. The dynamic condition requires that the load q must be equal to the viscous traction jump across the mechanics:

(6) $$\begin{array}{c} q=\left(\tau_{out}-\tau_{in}\right)\cdot n. \end{array}$$

The problem is characterized by Reynolds number Re and the capillary number Ca:

(7) $$\begin{array}{cc} \; & Re=\frac{\rho DV_{max}^{\infty }}{\mu_{0}}, \end{array}$$

(8) $$\begin{array}{cc} \; & Ca=\frac{\mu_{0}{\dot{\gamma }}_{m}a}{G_{s}}=\frac{\mu_{0}V_{max}^{\infty }}{G_{s}}\frac{a}{4R}, \end{array}$$

where $V_{max}^{\infty }$ (2$V_{m}^{\infty }$) is the maximum plasma velocity in the absence of any cells and ${\dot{\gamma }}_{m}$ ($V_{m}^{\infty }/D$) is the mean shear rate. Especially for the human microcirculation, flow can be assumed as inertialess, and is represented by Re = 0.2 (corresponding to particle Reynolds number $Re_{p}\left(\rho {\dot{\gamma }}_{m}a^{2}/\mu_{0}=\left(a^{2}/\left(2D^{2}\right)\right)Re\right)\approx$ 0.007) in this study. Although such a finite but low Re accurately represents the capsule dynamics solved by the boundary integral method (BIM) in Stokes flow [3,24,33], we further tested a capsule deformation including large Ca (≥1) (see also Appendix A). The condition defined by Ca = 0.05 (and Re = 0.2) corresponds to a typical venular wall-shear rate of 333 s${}^{-1}$ [34], and Ca = 0.1 corresponds to an arteriolar wall-shear rate of 670 s${}^{-1}$ [35] if the surface shear elastic modulus is considered as $G_{s}$ = 4 μN/m. Increasing Re under constant Ca corresponds to increasing $G_{s}$, namely, a harder RBC. Unless otherwise specified, we show the results obtained with Re = 0.2 ($Re_{p}\approx$ 0.007).

### 2.2. Numerical Simulation

We used the D3Q19 LBM [36] coupled with the finite element method (FEM) [37]. Based on the virtual work principle, the above strong form (2) can be rewritten in weak form as

(9) $$\int_{S}\hat{u}\cdot qdS=\int_{S}\hat{\epsilon }:TdS,$$

where $\hat{u}$ and $\hat{\epsilon }=\left(\nabla_{s}\hat{u}+\nabla_{s}{\hat{u}}^{T}\right)$ are the virtual displacement and virtual strain, respectively. The FEM is used to solve Equation (9) and obtain the load q acting on the membrane [37]. FEM and LBM were coupled by the immersed boundary method [38]. All procedures were fully implemented on a GPU to accelerate the numerical simulation. The flow was driven by a pressure gradient. Periodic boundary conditions were imposed on the inlet and outlet (*z*-direction). No-slip conditions were employed for the walls (*x*- and *y*-directions). The mesh size of the LBM for the fluid solution was set to be 250 nm, and that of the finite elements describing the membrane was approximately 250 nm (an unstructured mesh with 5120 elements was used for the FEM). This resolution has been shown to successfully represent single- and multicellular dynamics [24]; further, the results of multicellular dynamics are not changed by using twice the resolution for both the fluid and membrane meshes [24].

### 2.3. Analysis

To quantify the effect of the radial position of the RBC centroid and the deformed cell shape on fluid flow, the power (or energy dissipation) associated with membrane deformations is considered, and is given by

(10) $$\begin{array}{cc} \delta W_{mem}\; & =\int \hat{q}\cdot \left(v^{\left(m\right)}-V^{\infty }\left(r\right)\right)dS,\\ \to \delta W_{mem}^{*}\; & =\frac{2}{\mu_{0}DV_{max}^{\infty 2}}\int {\hat{q}}^{*}\cdot \left(v^{(m)*}-V^{\infty *}\left(r\right)\right)dS^{*}, \end{array}$$

(11) $$\begin{array}{cc} \; & =\frac{2}{\mu_{0}DV_{max}^{\infty 2}}\int \left[{\hat{q}}_{x}^{*}v_{x}^{(m)*}+{\hat{q}}_{y}^{*}v_{y}^{(m)*}+{\hat{q}}_{z}^{*}\left(v_{z}^{(m)*}-V_{z}^{\infty *}\left(r\right)\right)\right]dS^{*}, \end{array}$$

where $V^{\infty }\left(r\right)=\left(0,0,V_{max}^{\infty }\left[1-{\left(r/R\right)}^{2}\right]\right)$ is the fluid flow velocity without cells, $\hat{q}$ is the load acting on the membrane and includes the contribution of bending rigidity, *r* is the membrane distance from the channel center, $v^{\left(m\right)}$ is the interfacial velocity of the membrane, and *S* is the membrane surface area. Here, nondimensional variables are defined as ${\hat{q}}^{*}=\hat{q}/\left(\mu_{0}{\dot{\gamma }}_{m}\right)$, $v^{(m)*}=v^{\left(m\right)}/V_{max}^{\infty }$, $V^{\infty *}=V^{\infty }/V_{max}^{\infty }$ and $S^{*}=S/D^{2}$.

## 3. Results

### 3.1. Effect of Capillary Number Ca on RBC Shapes

**Figure 2:**
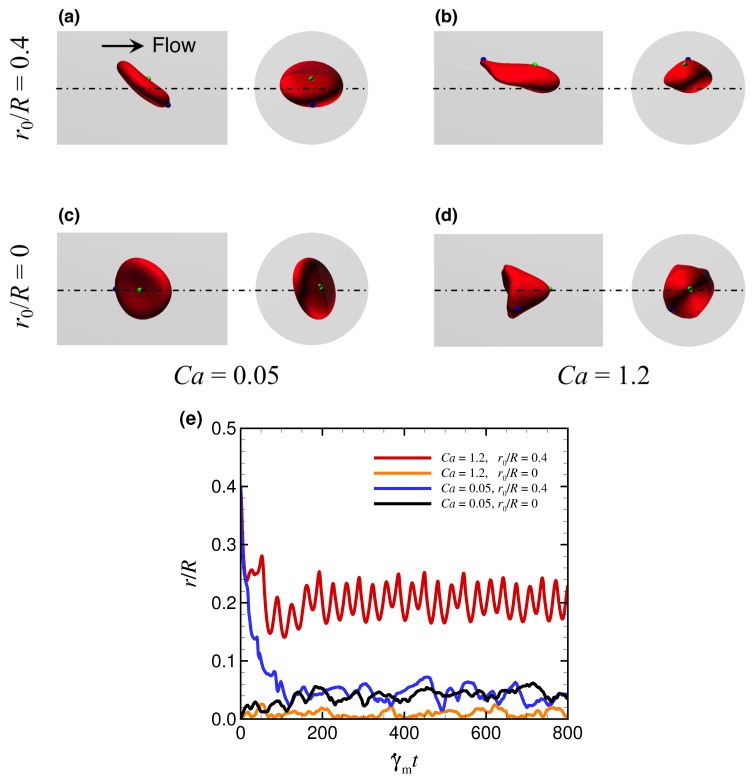
Snapshots of flowing RBCs in steady state (${\dot{\gamma }}_{m}t$ = 800) for (**a**,**c**) Ca = 0.05 (see also Video S1 and S3) and (**b**,**d**) Ca = 1.2 (see also Video S2 and S4). The right side is the axial view and the left side is the lateral view; the flow direction is from left to right. For each Ca, the initial position of the RBC centroid is set to be (**c**,**d**) $r_{0}/R$ = 0 and (**a**,**b**) $r_{0}/R$ = 0.4. (**e**) Time history of the radial position of the RBC centroid r/R for different Ca and $r_{0}/R$. The results were obtained with λ = 5.

**Figure A2:**
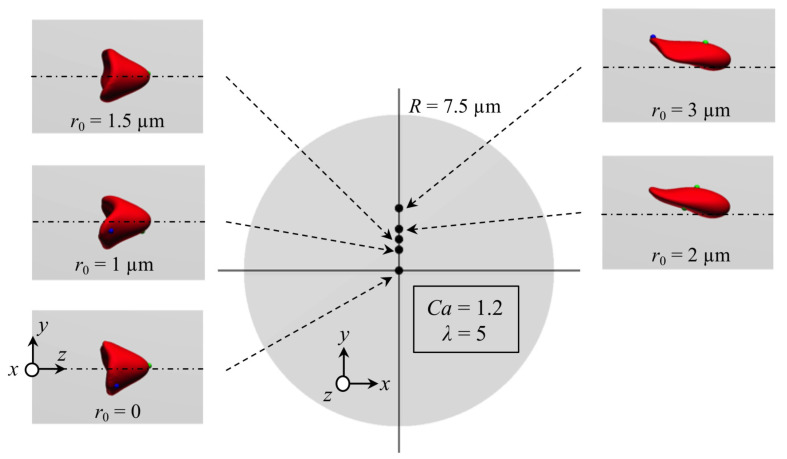
Snapshots of flowing RBCs in steady state at Ca = 1.2 for different initial positions $r_{0}$ (0, 1 μm, 1.5 μm, 2 μm, and 3 μm). The results were obtained with Re = 0.2 and λ = 5.

First, we investigated the behavior of RBCs with a viscosity ratio λ = 5 for different Ca and different initial radial positions of the RBC centroid $r_{0}$. Figure 2a–d show examples of snapshots of flowing RBCs in a steady state (${\dot{\gamma }}_{m}t$ = 800, see also Videos S1–S4). Initially off-centered RBCs ($r_{0}/R$ = 0.4) subjected to the lowest Ca (0.05) gradually migrated toward the channel center and exhibited a non-tank-treading (non-TT) discoidal shape, as shown in Figure 2a (see also Video S1). This occurred even when the initial position was set to be the channel center (Figure 2c, see also Video S3). Initially off-centered RBCs that were subjected to the highest Ca (1.2) exhibited a TT slipper shape [5,13], as shown in Figure 2b (see also Video S2). Since initially centered RBCs exhibited a non-TT parachute shape (Figure 2d, see also Video S4), the stable shape of RBCs subjected to a higher Ca depends on the initial position of the RBC centroid, which qualitatively agrees with previous experimental and numerical results in the rectangular microchannel [5]. Our numerical results further showed that a non-TT discoidal shape was observed for Ca≤ 0.1, which shifted to a non-TT parachute shape for 0.4 ≤Ca≤ 0.8, regardless of initial position. Bistable shapes (non-TT parachute shape and TT slipper shape) were only observed for Ca = 1.2. Note that at Ca = 1.2, a non-TT parachute shape was observed for $r_{0}\leq$ 1.5 μm (i.e., $r_{0}/R\leq$ 0.2), while a TT slipper shape was observed for $r_{0}\geq$ 2 μm (i.e., $r_{0}/R\geq$ 0.27) (see also Figure A2 in Appendix B).

**Figure A3:**
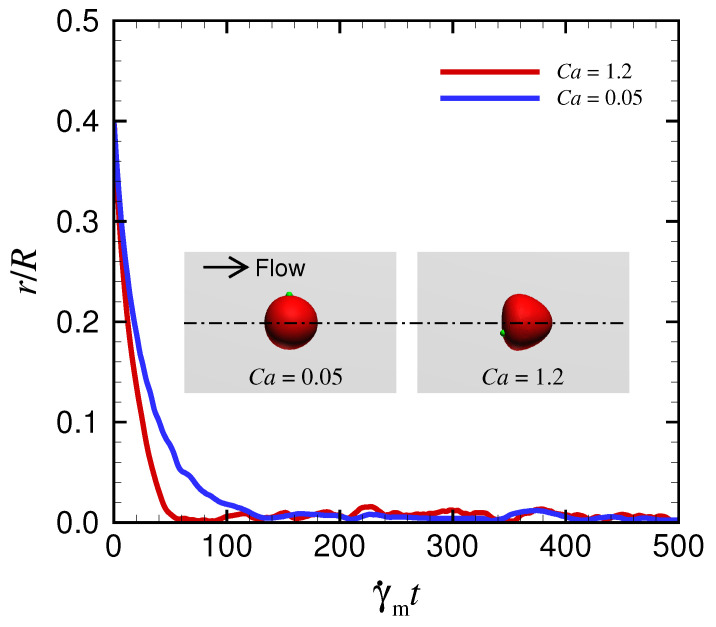
Time history of radial position of capsule centroid for different Ca (0.05 and 1.2), where the insets are the snapshots of flowing spherical capsule at steady state for each Ca (see also Videos S5 and S6). The results were obtained with Re = 0.2 and λ = 5.

Figure 2e shows the time history of the radial position of each RBC centroid *r*, normalized by the channel radius *R*. Spherical capsules uniformly exhibited axial migration independent of Ca (Figure A3 in Appendix C, see also Videos S5 and S6), which is consistent with classical principles regarding the axial migration of deformable spherical particles [8]; however, RBCs exhibited well-centered and off-centered migration depending on Ca and the initial radial position. In this study, we hereafter define axial migration as having an order of magnitude O(〈r〉/R)≤ 10${}^{-2}$, and nonaxial migration as having an order of magnitude O(〈r〉/R)> 10${}^{-2}$, where 〈·〉 denotes the time average. The time-averaging was performed for data after ${\dot{\gamma }}_{m}t$ = 200 based on Figure 2e. The non-TT discoidal shape, obtained with Ca = 0.05 (Figure 2a,c), flowed near the channel axis (blue and black lines in Figure 2e). The TT slipper shape, obtained with Ca = 1.2 and $r_{0}/R$ = 0.4 (Figure 2b), exhibited nonaxial migration with temporal fluctuations (red line in Figure 2e). The non-TT parachute shape, obtained with Ca = 1.2 and $r_{0}/R$ = 0 (Figure 2d), showed stable flow near the channel axis (orange line in Figure 2e). Note that the equilibrium radial position of each RBC was independent of its initial orientation, which was randomly determined (data is not shown).

**Figure 3:**
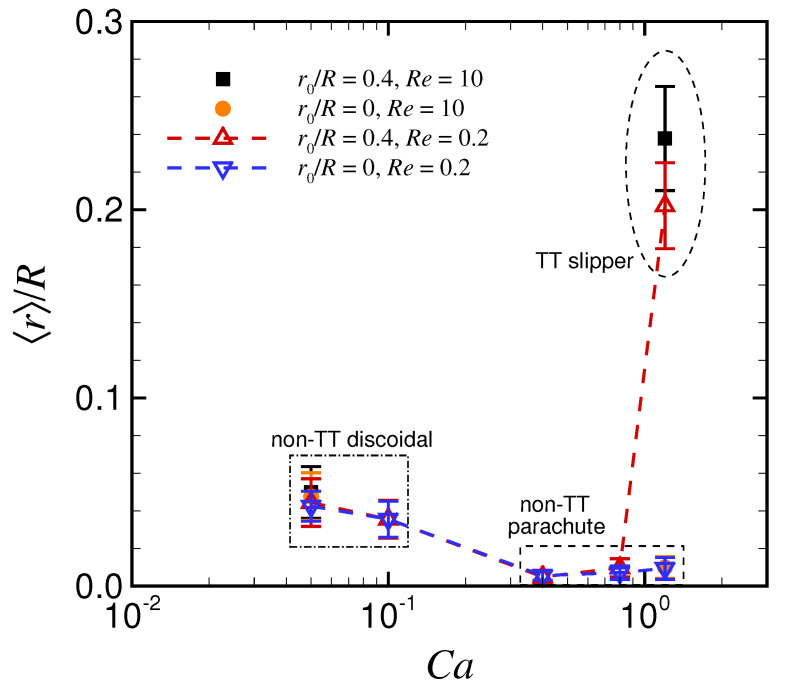
Time average of the radial position of the RBC centroid 〈r〉/R as a function of Ca for initial position $r_{0}/R$ = 0 (triangles) and $r_{0}/R$ = 0.4 (inverse triangles), where 〈·〉 denotes time average. The error bars represent standard deviations on the time axis. The results at Re = 10 for low Ca (0.05) and high Ca (1.2) are also plotted, with black squares for $r_{0}/R$ = 0.4 and orange circles for $r_{0}/R$ = 0. The results were obtained with λ = 5.

Figure 3 shows the time average of the radial position of the RBC centroid as a function of Ca, where the error bars represent standard deviations of the time axis. RBCs exhibited axial or nonaxial migration depending on Ca and the initial radial position. For relatively low Ca≤ 0.1, RBCs with a non-TT discoidal shape were located slightly away from the channel center regardless of initial position. For 0.4 ≤Ca≤ 0.8, RBCs with a non-TT parachute shape were much closer to the channel axis. For larger Ca (1.2), RBCs exhibited axial migration with a non-TT parachute shape and nonaxial migration with a TT slipper shape. Hence, the equilibrium radial position depends on the stable deformed RBC shape, which in turn, is related to Ca and the initial position. Note that aforementioned stable deformed shapes (i.e., non-TT discoidal, non-TT parachute, and TT slipper shapes) remained consistent even at high Re = 10, as did the equilibrium position (orange/black dots at Ca = 0.05 and Ca = 1.2).

**Figure 4:**
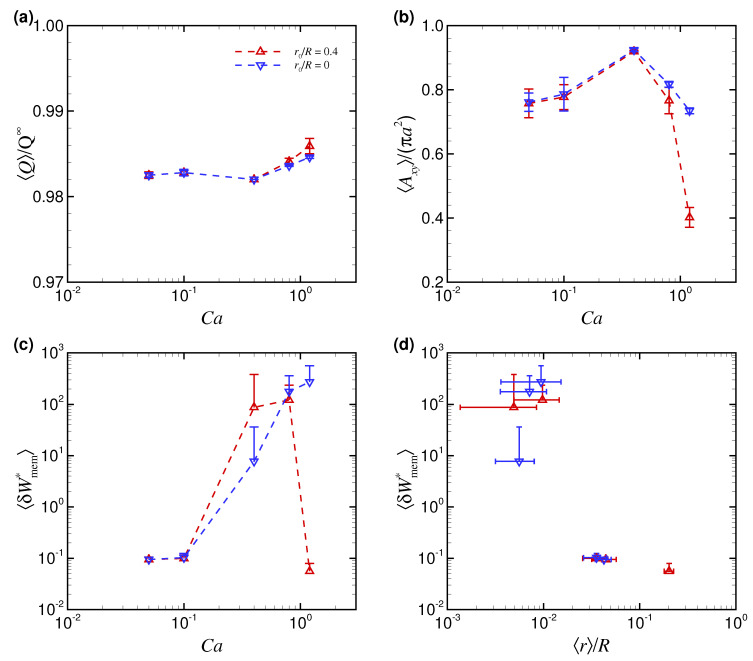
(**a**) Time average of the volumetric flow rate $\langle Q\rangle /Q^{\infty }$, (**b**) projected area of the RBC to the cross-sectional area of the channel (*x*-*y* plane) $\langle A_{xy}\rangle /\left(\pi a^{2}\right)$, and (**c**) powers associated with membrane deformations $\langle \delta W_{mem}^{*}\rangle$ as a function of Ca for different initial positions $r_{0}/R$ (0 and 0.4). (**d**) Replotted data of $\langle \delta W_{mem}^{*}\rangle$ as a function of equilibrium radial position 〈r〉/R for different Ca. The error bars represent standard deviations on the time axis. The results were obtained with λ = 5.

Figure 4a shows the time average of the volumetric flow rate 〈Q〉 with a RBC measured in simulations and normalized by the flow rate $Q^{\infty }$ ($\pi R^{2}V_{m}^{\infty }$) without a RBC as a function of Ca. The result also represents the change in flow resistance because the apparent viscosity is inversely proportional to the volumetric flow rate. Thus, the decrease of 〈Q〉 means the increase of the apparent viscosity. $\langle Q\rangle /Q^{\infty }$ was the largest with the TT slipper shape (red dot at Ca = 1.2 in Figure 4a)—i.e., the flow resistance was the smallest—because the projected area of the deformed RBC to the cross-sectional area of the channel (*x*-*y* plane) $A_{xy}$ was the smallest, as shown in Figure 4b, where $A_{xy}$ is normalized by the initial projected RBC area with maximal length, i.e., $\pi a^{2}$. Since fluid drag force can be described as a proportion of the projected area, the TT slipper shape with small projected area $A_{xy}$ leads to a smaller flow resistance than the other two shapes (non-TT discoidal/parachute shape) (Figure 4a). A reduction in the flow resistance, accompanied with the transition from non-TT discoidal/parachute shapes to TT slipper shapes with the increases of Ca, is consistent with [13].

The powers associated with membrane deformations $\langle \delta W_{mem}^{*}\rangle$ are shown in Figure 4c. The results of $\langle \delta W_{mem}^{*}\rangle$ were not always correlated with those of 〈Q〉 and $\langle A_{xy}\rangle$. $\langle \delta W_{mem}^{*}\rangle$ obtained with $r_{0}/R=0.4$ tended to increase for Ca = 0.8, but abruptly decreased for further large Ca (>0.8) (red dots in Figure 4c). $\langle \delta W_{mem}^{*}\rangle$ obtained with $r_{0}/R=0$ tended to increase, at least for Ca≤ 1.2 (blue dots in Figure 4c). Since it was expected that $\langle \delta W_{mem}^{*}\rangle$ would represent a more precise membrane load state than 〈Q〉 or $\langle A_{xy}\rangle$, we replotted $\langle \delta W_{mem}^{*}\rangle$ as a function of 〈r〉/R, as shown in Figure 4d. The result reflected the finding that $\langle \delta W_{mem}^{*}\rangle$ was not affected by the initial position of Ca, but instead, was large for the near-center position with 〈r〉/R≤ 10${}^{-2}$ and small for the off-centered position with 〈r〉/R> 10${}^{-2}$ (Figure 4d).

### 3.2. Effect of Viscosity Ratio λ on RBC Shapes

**Figure 5:**
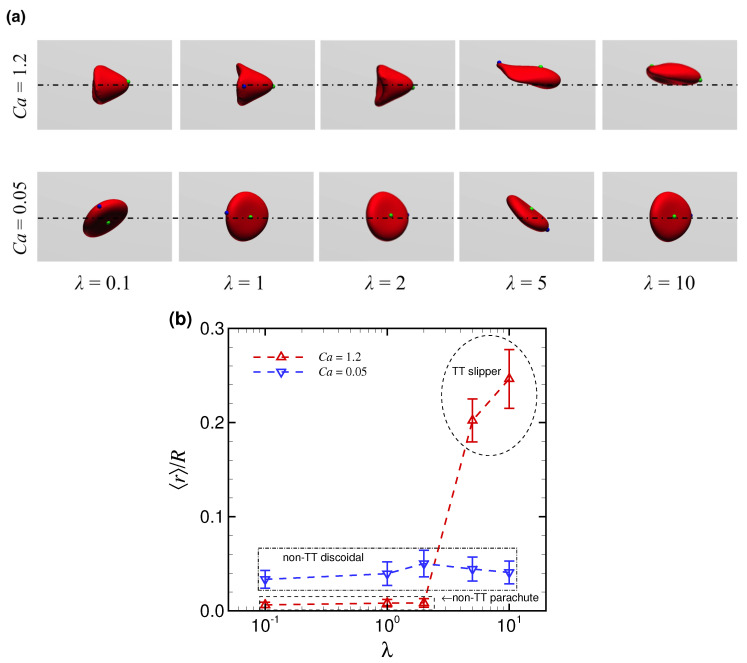
(**a**) Snapshots of flowing RBCs in steady state at Ca = 0.05 (bottom row) and Ca = 1.2 (top row) for different λ. (**b**) Time average of the radial position of the RBC centroid 〈r〉/R as a function of λ for Ca = 0.05 (blue inverse triangles) and Ca = 1.2 (red triangles). The results were obtained with an initial off-centered position at $r_{0}/R$ = 0.4.

Next, we investigated the effect of viscosity ratio λ on the behavior of RBCs. Figure 5a shows snapshots of flowing RBCs subjected to low and high Ca in steady state for different λ. The simulations were started from the off-centered position at $r_{0}/R$ = 0.4. RBCs subjected to low Ca (0.05) uniformly exhibited the non-TT discoid shape regardless of λ (bottom row in Figure 5a). In contrast, the stable shape of RBCs subjected to high Ca (1.2) changed from non-TT parachute shape for λ≤ 2 to TT slipper shape for λ≥ 5 (top row in Figure 5a).

Figure 5b shows the time average of the radial position of the RBC centroid 〈r〉/R as a function of λ. As described previously in Figure 3, non-TT parachute shapes, obtained with high Ca (1.2) and λ≤ 2, approached the channel axis much more closely than the non-TT discoidal shape obtained with low Ca (0.05). The TT slipper shape, which was only found for high Ca (1.2) and λ≥ 5, clearly exhibited nonaxial migration (i.e., $O\left(\langle r\rangle /R\right)>10^{-2}$). Furthermore, comparing the results of 〈r〉/R between λ = 5 and λ = 10, a higher viscosity ratio allowed RBCs to be positioned away from the channel center (Figure 5b).

**Figure 6:**
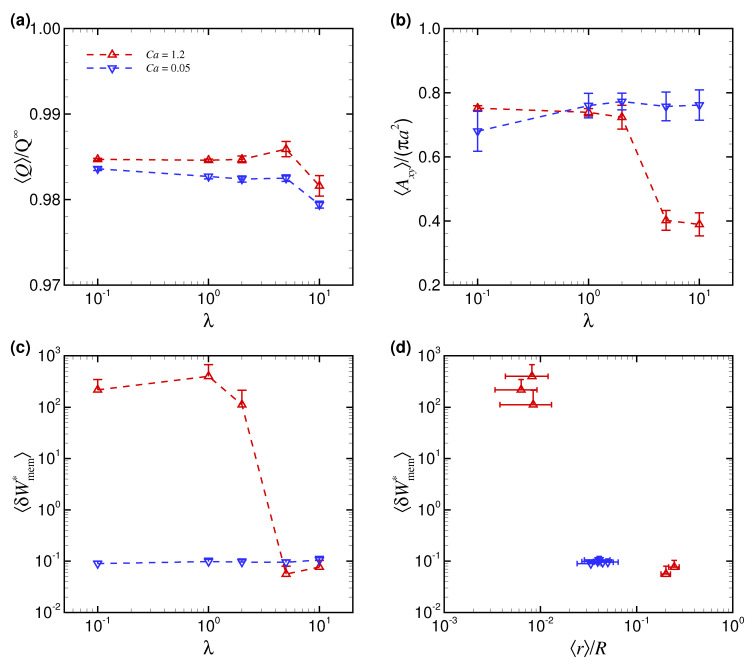
(**a**) Time average of the volumetric flow rate $\langle Q\rangle /Q^{\infty }$, (**b**) projected area of the RBC $\langle A_{xy}\rangle /\left(\pi a^{2}\right)$, and (**c**) powers associated with membrane deformations $\langle \delta W_{mem}^{*}\rangle$ as a function of λ for different Ca (0.05 and 1.2). (**d**) Replotted data of $\langle \delta W_{mem}^{*}\rangle$ as a function of equilibrium radial position 〈r〉/R for different λ. The results were obtained with an initial off-centered position $r_{0}/R$ = 0.4.

Figure 6a shows the time average of the volumetric flow rate 〈Q〉 as a function of λ. The non-TT discoidal shape, obtained with Ca = 0.05, was associated with a near-center position (Figure 5b) and a relatively large projected area $A_{xy}$ (Figure 6b), resulting in large flow resistance, i.e., small $\langle Q\rangle /Q^{\infty }$ (Figure 6a). Although the non-TT parachute shape obtained with Ca = 1.2 and λ≤ 2 was also associated with a large $A_{xy}$, $\langle Q\rangle /Q^{\infty }$ was larger than with the non-TT discoidal shape (Figure 6a). The values of $A_{xy}$ for the TT slipper shape, which was obtained with Ca = 1.2 and λ≥ 5, were smaller than for the other two shapes (Figure 6b). Further, with the TT slipper shape, the value of $\langle Q\rangle /Q^{\infty }$ was relatively large except for λ = 10 (Figure 6a). These results suggest that the projected area $A_{xy}$ is not always correlated with flow resistance represented by $\langle Q\rangle /Q^{\infty }$, and hence, a more precise description of membrane dynamics is necessary to better understand the relationship between stable shapes and equilibrium radial positions.

Figure 6c shows the powers associated with membrane deformations $\langle \delta W_{mem}^{*}\rangle$ as a function of λ. The results of $\langle \delta W_{mem}^{*}\rangle$ at Ca = 0.05 consistently had a relatively small order of magnitude, while those at Ca = 1.2 started to decrease as λ increased to be >1 (Figure 6c). These trends are consistent with those in the equilibrium position 〈r〉/R described in Figure 5b. Although the results of $\langle \delta W_{mem}^{*}\rangle$ did not correlate with those of $\langle Q\rangle /Q^{\infty }$, as previously described in Figure 4a,c, they did correlate well with the equilibrium position 〈r〉/R regardless of λ and Ca, as shown in Figure 6d.

## 4. Discussion

**Figure 7:**
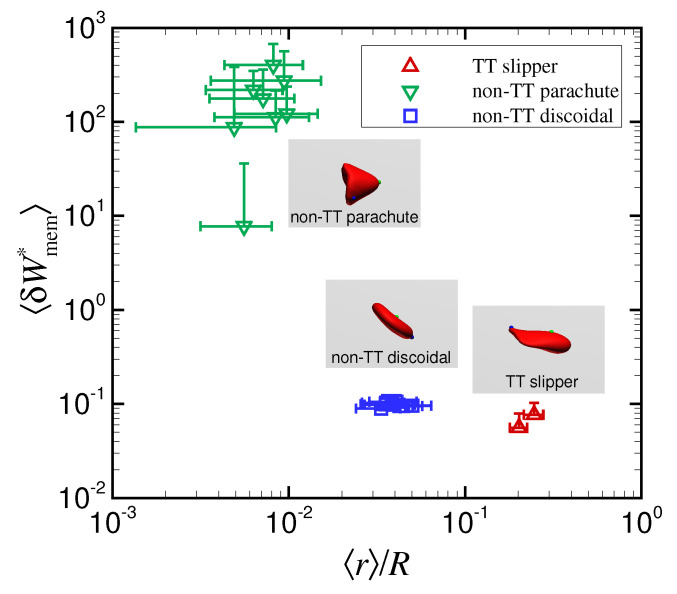
Powers associated with membrane deformations $\langle \delta W_{mem}\rangle$ as a function of equilibrium radial position 〈r〉/R for different shapes. The results are replotted from Figure 4d and Figure 6d.

Various numerical models have been used to systematically investigate the behavior of a single RBC at low Re in microchannels whose scale is comparable to the cell size [5,12,13]. For instance, Fedosov et al., (2014) performed simulations of RBC behavior for a fixed viscosity ratio λ (1) in circular microchannels and showed a phase diagram of stable RBCs as a function of shear rates and size ratios in the range of 0.3 <d/D< 0.8 [13]. Guckenberger et al., (2018) also performed simulations of RBCs for a viscosity ratio λ = 5 in a rectangular microchannel of width *W* = 12 μm and height *H* = 10 μm (i.e., d/H = 0.8 and d/W = 0.67), and showed a phase diagram of stable RBCs as a function of cell velocity and initial position [5]. However, it has not yet been fully determined how these shapes at equilibrium position contribute to fluid flow. In this study, we further investigated energy expenditure due to membrane deformation of RBCs in a circular microchannel with a diameter of *D* = 15 μm, i.e., d/D = 0.53, and found that it correlated well with the equilibrium position of the RBC regardless of Ca, λ, and the initial position of the RBC centroid (Figure 4d and Figure 6d). The results are summarized in Figure 7, which consists of replotted data from Figure 4d and Figure 6d. The results suggest that the equilibrium radial position of the RBC centroid is determined by the stable deformed shape, due to different energy expenditures associated with various membrane deformations. The non-TT parachute shape allows the RBC to approach the channel axis with an order of magnitude $O\left(\langle r\rangle /R\right)∼10^{-2}$, while the radial position of the non-TT discoidal shape is shifted slightly away from the channel center with $10^{-2}<\langle r\rangle /R<10^{-1}$ (Figure 7). The TT slipper shape always demonstrates nonaxial migration with $\langle r\rangle /R>10^{-1}$ (Figure 7). This shape was only observed in limited conditions: Ca = 1.2, λ≥ 5, and an initial off-centered position $r_{0}/R$ = 0.4. Such high Ca (1.2) corresponds to a higher wall shear rate in a circular channel ${\dot{\gamma }}_{wall}\left(8{\dot{\gamma }}_{m}\right)$ = 8 × 10${}^{3}$ s${}^{-1}$, which is over 10 times greater than human arterial wall shear rates [35]. Therefore, it is expected that the TT slipper shape with an off-centered position will be found in in vitro systems with artificially high shear rates except for pathological vascular regions, e.g., arterial stenosis [39]; hence, this shape may be a hallmark in cell sorting techniques using microfluidics. Furthermore, at high Ca (1.2), the parachute shape was found instead of the TT slipper shape for λ≤ 2 (Figure 5a). Thus, the parachute shape may also be useful as an indicator for identifying cytoplasmic viscosity. Since we set the channel diameter to be 15 μm, it would be interesting to study how the off-centered position 〈r〉/R depends on stable deformed shape changes in larger microtubes.

The motion of a TT slipper shape in this study may be redefined as a snaking motion (periodic oscillation of the shape in the form of a snake motion), according to previous numerical analyses using a 2D vesicle model [40,41,42]. However, in this study, we focused on stable deformed RBC shapes, and did not rigorously differentiate between TT slipper shape and snaking motion, following from previous numerical studies [5,43]. Although we have shown that our numerical models successfully reproduce not only the deformation of single RBC but also the thickness of the cell-depleted peripheral layer due to multicellular interactions [24], comparisons in flowing RBC shapes between numerical results and experimental observations in microtubes have not yet been conducted, which is our future study. As in many numerical studies, e.g., [5,43], we also neglected membrane viscosity, which has been introduced only in a few continuum model analyses [44,45,46]. Hence, it would be also interesting to study how such fluid deformable surfaces changes the stable deformed RBC shape relative to a pure elastic membrane. Although we performed simulations for a wide range of Ca and λ, we are unsure what factors cause RBCs to adopt a stable shape under specific equilibrium positions. Considering the finding that flow resistance characterized by the volumetric flow rate $\langle Q\rangle /Q^{\infty }$ is not always described by the projected area of the RBC to the cross-sectional area of the channel $A_{xy}$ (Figure 4b and Figure 6b), membrane dynamics need to be more precisely investigated to clarify this problem. In the future, we will report the precise mechanical characteristics of the stable deformed RBC shapes, as well as the relationship between these stable shapes, the equilibrium radial positions of RBCs, and the membrane load.

## 5. Conclusions

We numerically investigated the dynamics of translating RBCs in a circular microchannel with a diameter of 15 μm for different capillary numbers Ca and viscosity ratios λ. The flow was assumed to be almost inertialess. Our results demonstrated that the presence of axial or nonaxial migration depends on the stable deformed RBC shapes, and that the equilibrium radial position of the RBC centroid correlated well with the energy expenditure associated with different membrane deformations. The non-TT parachute shape, obtained with high Ca and low λ, allowed RBCs to approach the channel axis, while the non-TT discoidal shape, obtained with low Ca, shifted the radial position of RBCs slightly away from the channel center. The TT slipper shape, obtained with high Ca and high λ, was always accompanied by obvious nonaxial migration. The energy expenditure decreased in the following order: Non-TT parachute shape, non-TT discoidal shape, and TT slipper shape. In the near future, we will examine the shape stability of deformed RBCs in more details to clarify precise mechanical characteristics of the stable shapes, and report the relationship between these stable shapes, the equilibrium radial position of RBCs, and the membrane load.

## Figures and Tables

**Figure A1:**
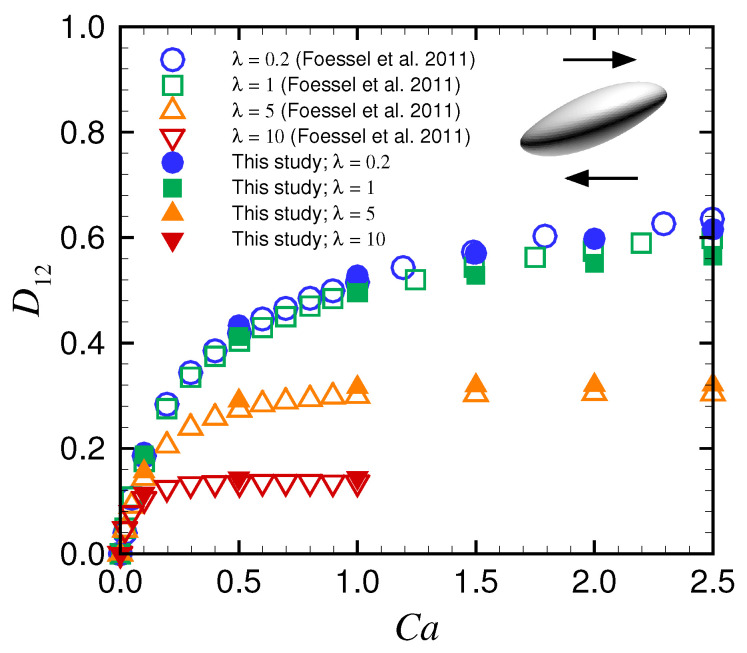
Time-averaged Taylor parameters $D_{12}$ of an SK spherical capsule as a function of Ca for different viscosities λ (0.2, 1, 5, and 10); previous numerical results of Foessel et al. [48] are also displayed. The inset represents a tank-treading spherical capsule at Ca = 1.0 and λ = 1. The results were obtained with $Re_{p}$ = 0.2.

## Supplementary Materials

The following materials are available online at https://www.mdpi.com/article/10.3390/mi12101162/s1. Video S1: Non-TT discoidal shape, obtained with Ca = 0.05 and $r_{0}/R$ = 0.4. Video S2: TT slipper shape, obtained with Ca = 1.2 and $r_{0}/R$ = 0.4. Video S3: Non-TT discoidal shape, obtained with Ca = 0.05 and $r_{0}/R$ = 0. Video S4: Non-TT parachute shape, obtained with Ca = 1.2 and $r_{0}/R$ = 0. Videos S5 and S6: Numerical results of a spherical capsule initially placed at $r_{0}/R$ = 0.4 for Ca = 0.05 and Ca = 1.2, respectively. These numerical results were obtained with Re = 0.2 and λ = 5.

## Abbreviations

The following abbreviations are used in this manuscript:

RBC	Red blood cell
LBM	Lattice-Boltzmann method
FEM	Finite element method
IBM	Immersed boundary method
GPU	Graphics processing unit
(non-)TT motion	(non-)tank-treading motion

## Appendix A. Deformation of a Spherical Capsule

To validate our numerical models, we tested the deformation of a single spherical capsule for different Ca (≤2.5) and different λ (0.2, 1, 5, and 10) under shear flow in a cubic domain of size with 8a×8a×8a. Particle Reynolds number was set to be $Re_{p}$ ($\rho \dot{\gamma }a^{2}/\mu_{0}$) = 0.2. The shear flow is drive by moving the top and bottom walls located at y=±H/2 (H=8a). Periodic boundary conditions are imposed on the two homogeneous directions (*x* and *z* directions). The resolutions of the fluid and membrane meshes are the same as in the analysis above. The capsule deformation is quantified by the Taylor parameter defined as $D_{12}=\left|a_{1}-a_{2}\right|/\left(a_{1}+a_{2}\right)$, where $a_{1}$ and $a_{2}$ are the lengths of the semimajor and semiminor axes of the deformed capsule, and are obtained from the eigenvalues of the inertia tensor of an equivalent ellipsoid approximating the deformed capsule [47]. Time average starts after the nondimensional time $\dot{\gamma }t$ = 60 to reduce the influence of the initial conditions, and continues to $\dot{\gamma }t\geq$ 100. Our numerical results are compared with previous numerical results obtained with the BIM [48]. For reasonable comparison with previous numerical studies [48], the same parameters are considered and the membrane is modeled with the SK law (1) with the area dilation modulus *C* = 1 and without bending resistance. Figure A1 shows that our numerical results are in good agreement with those of [48].

## Appendix B. Effect of Initial Position of RBC on Stable Deformed Shapes

To clarify the reproducibility of the stable shapes of a deformed RBCs, we investigated the effects of the initial position of RBC centroid (0 $<r_{0}/R<$ 0.4) as a potential perturbation. Figure A2 shows snapshots of flowing RBCs in steady state at Ca = 1.2 for different initial positions $r_{0}$. Initially 1.5-μm-off-centered RBCs (i.e., $r_{0}/R\leq$ 0.2) attained a non-TT parachute shape, while initially 2-μm-off-centered RBCs (i.e., $r_{0}/R\geq$ 0.27) attained a TT slipper shape (Figure A2). Although the effect of the initial position on the final stable shape would depend on Ca, more strict examinations about shape stability will be discussed in our future study.

## Appendix C. Behavior of a Spherical Capsule in a Circular Channel

We preliminarily tested the behavior of a spherical capsule with the same radius as an RBC (i.e., *a* = 4 μm) at Re = 0.2, and confirmed that an initially off-centered sphere ($r_{0}/R$ = 0.4) exhibited axial migration for both low Ca (0.05) and high Ca (1.2), as shown in Figure A3 (see also Videos S5 and S6). The speed toward the channel center was slightly higher for a more deformable capsule with Ca = 1.2 than for a less deformable capsule with Ca = 0.05 (Figure A3). This result was consistent with experimental study using a circular channel at low Re (≪1) [8].
